# Upon the Destruction of the Puerperal Miasma in Lying-In Hospitals

**Published:** 1853-05

**Authors:** 


*Upon the Destruction of the Puerperal Miasma in Lying-In Hospitals.* By Dr. Busch.—The means employed by the author consist in heating the room to a high degree with dry air. This is effected by round iron stoves placed in the centre of the room, and connected with the chimney by metal tubes. The heat can be raised to 50.60° R. (about 155° F.) This must be kept up for two days, during which time all furniture and utensils are to remain in the room.

In March, 1851, puerperal fever invaded the Berlin Lying-in Hospital with remarkable severity; nearly all the patients suffered, and the Institution was closed for six weeks, during which time there was the most careful ventilation and purification. These means proved insufficient. Upon the re-opening of the hospital, all the new patients became attacked by the disease a few days after delivery. Then the author tried the plan here detailed in every room in the house. The effect was surprising; no fresh attack occurred during the whole summer. The same measures were adopted some time afterwards, and with the same success. —*London Med. Times, from N. Ztschr. für Gebwitsk.*

